# A Double-Blinded, Randomized, Vehicle-Controlled Study of the Efficacy of Moisturizer Containing Licochalcone A, Decanediol, L-Carnitine, and Salicylic Acid for Prevention of Acne Relapse in Asian Population

**DOI:** 10.1155/2020/2857812

**Published:** 2020-10-16

**Authors:** Kanokvalai Kulthanan, Suphattra Trakanwittayarak, Papapit Tuchinda, Leena Chularojanamontri, Pichaya Limphoka, Supenya Varothai

**Affiliations:** Department of Dermatology, Faculty of Medicine Siriraj Hospital, Mahidol University, Bangkok, Thailand

## Abstract

Many topical agents are available for treating the acute phase of acne; however, few agents have been proven beneficial during the maintenance phase. *Objective.* To evaluate the efficacy and safety of moisturizer containing licochalcone A, 1,2-decanediol, L-carnitine, and salicylic acid during the maintenance phase of mild to moderate acne in Thai patients. *Methods.* One hundred and ten patients with mild to moderate acne vulgaris were initially treated with a fixed combination of adapalene 0.1%/benzoyl peroxide 2.5% gel once daily for 8 weeks. Fifty patients who achieved at least 50% reduction in lesion counts or at least a 2-grade improvement in the Investigator's Global Assessment (IGA) grade from baseline were enrolled in the maintenance phase, which was an investigator-masked, left-right comparison, randomized, controlled, intraindividual study. Moisturizers with and without the active study ingredients were applied twice a day to each side of the face, respectively, for 12 weeks. Assessments included acne lesion counts, acne severity by IGA scoring, skin bioengineering measurements, and skin tolerability as assessed by both patient and physician. *Results.* The treatment group had a significant reduction in the mean counts of noninflammatory, inflammatory, and total lesions compared to the vehicle group at week 12 and also between baseline and week 12. There was no significant difference in the mean scores for skin dryness, stinging/burning, or pruritus at any time point between groups. *Conclusions.* Moisturizer containing licochalcone A, 1,2-decanediol, L-carnitine, and salicylic acid reduced acne lesions and prevented the development of new lesions during the maintenance phase. This trial is registered with ClinicalTrials.gov registration no. NCT04002024.

## 1. Introduction

Acne vulgaris is a common dermatological condition with a prevalence up to 85% among adolescents [1]. The four main mechanisms that lead to acne are abnormal keratinization, excessive sebum production, proliferation of *Cutibacterium acne (C. acnes)*, and inflammation [2]. The current treatments for acne are based on acne severity, which is categorized as mild, moderate, or severe [3]. Patients with mild disease are treated with topical regimens, including retinoids, benzyl peroxide, and/or antibiotics, and systemic treatments are added for patients with moderate to severe acne [1, 4]. Since acne is considered to be a persistent and relapsing inflammatory disease, a long-term maintenance therapy should be considered [5]. Antibiotic monotherapy, either topical or systemic therapy, was discouraged for maintenance therapy due to the development of *C. acnes* antibiotic resistance [4]. Topical retinoids are recommended as a mainstay treatment during the maintenance phase [6]. However, there are various cutaneous side effects, such as erythema, dryness, itching, stinging, and photosensitivity. Among topical retinoids, adapalene was reported to cause less skin irritation [7, 8]. As such, the side effects of topical retinoids may lead to a poor patient compliance and unsuccessful treatment [9].

Topical dermocosmetics with active ingredients that target the pathogenesis of acne have become a treatment of interest for maintenance therapy [10]. The roles of antiacne dermocosmetics as an adjunctive therapy may include prevention and maintenance, delivering synergistic effect, and management of side effects of antiacne medication [11]. The ideal dermocosmetics for acne should be noncomedogenic, hypoallergenic, nonirritating, and compatible with acne treatment [12]. Moisturizer containing active ingredients, such as licochalcone, 1,2-decanediol, L-carnitine, and salicylic acid, for the treatment of acne was reported to have beneficial effects when combined with standard treatments [13–16]. Licochalcone A has a highly effective anti-inflammatory effect [17], decanediol has antibacterial efficacy against *C. acnes* [18], and L-carnitine can reduce sebum production [19]. Salicylic acid at low concentration has mild comedolytic and corneolytic efficacy [20, 21].

The aim of this study was to evaluate the efficacy and safety of moisturizer containing licochalcone A, decanediol, L-carnitine, and 1% salicylic acid compared with placebo during the maintenance phase of mild to moderate acne vulgaris in Thai patients, which might represent Asian population.

## 2. Materials and Methods

### 2.1. Study Design

This 8-week open-label and 12-week, double-blind, randomized, vehicle-controlled study was conducted at the Department of Dermatology of the Faculty of Medicine Siriraj Hospital, Mahidol University, Bangkok, Thailand, during June 2019 to January 2020. This study was registered at the ClinicalTrials.gov website (registration no. NCT04002024), and it was approved by the Siriraj Institutional Review Board (SIRB) (COA no. Si 079/2019). All subjects provided written informed consent prior to entering the study.

### 2.2. Study Population

Male and female participants aged more than 18 years who were diagnosed as acne vulgaris by dermatologists were enrolled. Acne vulgaris was defined by polymorphic lesions of comedones, erythematous papules, pustules, and nodules on a seborrheic area. Patients who had acne on both cheeks with mild to moderate severity consistent with an Investigator's Global Assessment (IGA) [22] score of 2 or 3 were recruited. The exclusion criteria were patients with the following: (i) other types of acne, (ii) other active skin diseases on the face for the previous 2 weeks, (iii) receiving systemic treatment for acne within 4 weeks prior to the start of this study, (iv) history of allergic contact dermatitis to any of the ingredients in the test product, (v) history of adverse reactions to doxycycline, and/or (vi) pregnant or breastfeeding women. Eligible female subjects must have used any reliable contraceptive method besides oral contraceptive pills for at least 1 month prior to the start of this study and must have agreed to do the same for at least 6 months after the completion of the study.

### 2.3. Sample Size Calculation

In a study by Leyden et al. [23], 189 patients with moderate to moderately-severe acne vulgaris were enrolled to investigate the maintenance effect of 3 regimens (topical tazarotene, oral minocycline hydrochloride, or both) for treating patients with acne vulgaris. The improvement in patients receiving tazarotene and in patients receiving minocycline hydrochloride were adopted to calculate our sample size. In that study, the percentage difference in the mean inflammatory lesion count from baseline was −54.0 ± 25.1 and −66.0 ± 29.4 in the tazarotene and minocycline hydrochloride groups, respectively. Assuming a significance level of 0.05 and 80% power, 50 patients were required to detect a 1-point difference in the mean inflammatory lesion count, and a 2.5-point difference in the standard deviation (SD) between groups during the maintenance phase.

From studies by Gold et al. [24] and Poulin et al. [25] 53% of patients receiving doxycycline 100 mg and adapalene 0.1%/benzoyl peroxide 2.5% gel had at least 50% improvement after 12 weeks of treatment. Accordingly, if 50 patients were required for the maintenance phase, at least 100 patients would be required for the induction phase. To compensate for a dropout rate of 10% for any reason, a total of 110 patients were enrolled in the induction phase of this study.

### 2.4. Study Treatments

This study was divided into 2 phases—the induction phase and the maintenance phase. All patients were allowed to use only lipstick and their previously and regularly used face puffs after enrollment in the study. Other skin care products and other topical or systemic acne medications were prohibited throughout the study period. A specific cleanser (pH 5.5 mild cleanser without perfumes, hypoallergenic, and hypocomedogenic) was provided for all patients throughout the study period. Study moisturizer and placebo were provided for patients who met the criteria for inclusion in the maintenance phase.

During the induction phase (an 8-week open-label study), 110 patients with mild to moderate acne vulgaris, based on IGA scoring, were treated with a fixed combination of adapalene 0.1%/benzoyl peroxide 2.5% gel once daily for 8 weeks. In cases with moderate acne severity, 1-2 capsules of oral doxycycline per day were added for 1-2 months and then stopped prior to the maintenance phase. Patients who had at least 50% reduction in the number of acne vulgaris or at least a 2-grade improvement in IGA grading from baseline were enrolled in the maintenance phase.

For the maintenance phase (a 12-week double-blind, randomized, vehicle-controlled study), 50 patients were randomized into 5 blocks with 10 block sizes to apply moisturizer containing active ingredients (licochalcone A, decanediol, L-carnitine, and 1% salicylic acid) and placebo, which was identical to the moisturizer vehicle without the mentioned active ingredients. Patients were asked to regularly apply the moisturizer containing active ingredients and placebo (one on each side of the face) twice daily (one fingertip unit per each application) for 12 weeks.

**Figure 1:**
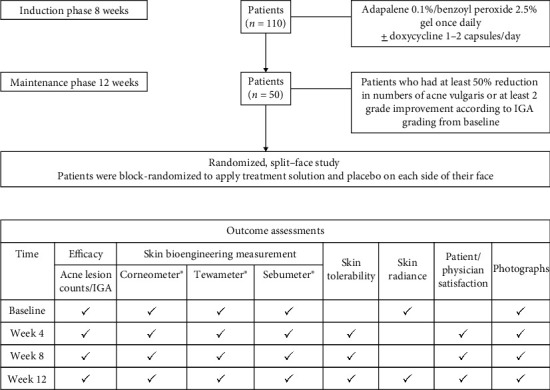
Flowchart of the study protocol. IGA: Investigator's Global Assessment.

The same investigator evaluated the skin condition of each patient during every visit. Patients were followed up every 4 weeks for 12 weeks. A flowchart of the study protocol is shown in Figure 1. Outcomes were assessed according to acne lesion counts, acne severity according to IGA scoring, skin bioengineering measurements, and skin tolerability as assessed by both patients and physicians. All data were analyzed at the end of the study.

### 2.5. Outcome and Methods of Evaluation

- *Efficacy Evaluation.* Efficacy rates were evaluated based on the count of noninflammatory acne lesions, inflammatory acne lesions, and total acne lesions and the IGA score at every visit during the maintenance phase. The severity of acne was graded according to IGA score [22], as follows: 0 = clear (no inflammatory or noninflammatory lesion), 1 = almost clear (rare noninflammatory lesions with no more than one small inflammatory lesion), 2 = mild (some noninflammatory lesions with no more than a few inflammatory lesions, but no nodular lesions), 3 = moderate (many noninflammatory lesions and may have some inflammatory lesions, but no more than one small nodular lesion), and 4 = severe (up to many noninflammatory and inflammatory lesions, but no more than a few nodular lesions). The maintenance rate was defined as the percentage of patients who maintained at least 50% improvement in the total lesion count between baseline of the maintenance phase and week 12 [26].
- *Skin Bioengineering Evaluation.* The following skin bioengineering measurements [27] were assessed: (i) water content of the stratum corneum by Corneometer® CM 825 (Courage-Khazaka, Cologne, Germany), (ii) transepidermal water loss (TEWL) by Tewameter® TM 300 (Courage-Khazaka, Cologne, Germany), and (iii) sebum amount by Sebumeter® SM 815 (Courage-Khazaka, Cologne, Germany) at baseline and weeks 4, 8, and 12. Before each set of measurements, participants were required to equilibrate in a closed standard environment with a constant temperature (20 ± 2°C) and humidity (45-55% relative humidity). All measurements were taken by the same investigator.
- *Skin Tolerability Evaluation.* Skin tolerability was evaluated by 5 parameters, including erythema, dryness, scaling, stinging/burning, and pruritus. All 5 parameters were assessed by the patient, and erythema, dryness, and scaling were evaluated by physicians. Results were recorded using a 4-point scale (0 = none, 1 = mild, 2 = moderate, and 3 = severe) on each side of the face at weeks 4, 8, and 12. Overall patient tolerance assessment was rated on a scale ranging from 0 to 3 (0 = poor, 1 = fair, 2 = good, and 3 = excellent) at weeks 4, 8, and 12.
- *Skin Radiance Evaluation.* Skin radiance was measured on each half-face at the beginning and end of the study by patients using a visual analog scale (VAS). The minimum score is 0 (no radiance at all), and the maximum score is 10 (most radiant) [28].
- *Satisfaction Evaluation*. Patient satisfaction was assessed at weeks 4, 8, and 12 using a 4-point scale (0 = not at all, 1 = mild, 2 = *m*oderate, and 3 = very satisfied) [29]. Overall patient satisfaction was assessed using a VAS at week 12. Similarly, physician satisfaction was evaluated at weeks 4, 8, and 12 using a 4-point scale (0 = poor, 1 = fair, 2 = good, and 3 = excellent), and overall physician satisfaction was assessed using a VAS at week 12.
- *Photographic Evaluation.* Standard facial photographs were taken to evaluate the clinical presentation of acne vulgaris. Digital ultraviolet (UV) fluorescence photography was performed using VISIA Complexion Analysis (software version 6.4.2, Canfield Scientific, Parsippany, NJ, USA). UV fluorescence photography demonstrates porphyrins that are produced by *C. acnes* and that become lodged in pores. Porphyrins demonstrate orange-red fluorescence under UVA light [30].

### 2.6. Statistical Analysis

PASW Statistics version 18 (SPSS, Inc., Chicago, IL, USA) was used for data analysis. All statistical tests were two-sided, and statistical significance was declared at a *p* value less than 0.05. A paired *t*-test was used to analyze changes in lesion counts, differences in skin bioengineering measurements, skin tolerability assessment, overall patient tolerance, skin radiance VAS score, patient/physician satisfaction, and patient/physician satisfaction VAS scores. The general linear model with repeated measure was used to analyze data within groups. McNemar's test was used to analyze the maintenance rate. Data are presented as the mean ± SD or number and percentage.

## 3. Results

### 3.1. Demographic and Clinical Characteristics

One hundred and ten patents were enrolled in the induction phase, and 50 patients (11 males, 39 females; mean age 28.2 years) who had at least 50% reduction in the number of acne lesions or at least a 2-grade improvement in IGA grading from baseline were included in the maintenance phase. None of the patients withdrew during the maintenance therapy. The demographic and clinical characteristics of these 50 patients are shown in Table 1. There were no significant differences in these characteristics or in skin bioengineering data between the treatment and placebo sides of the face.

### 3.2. Efficacy Evaluation

**Figure 2:**
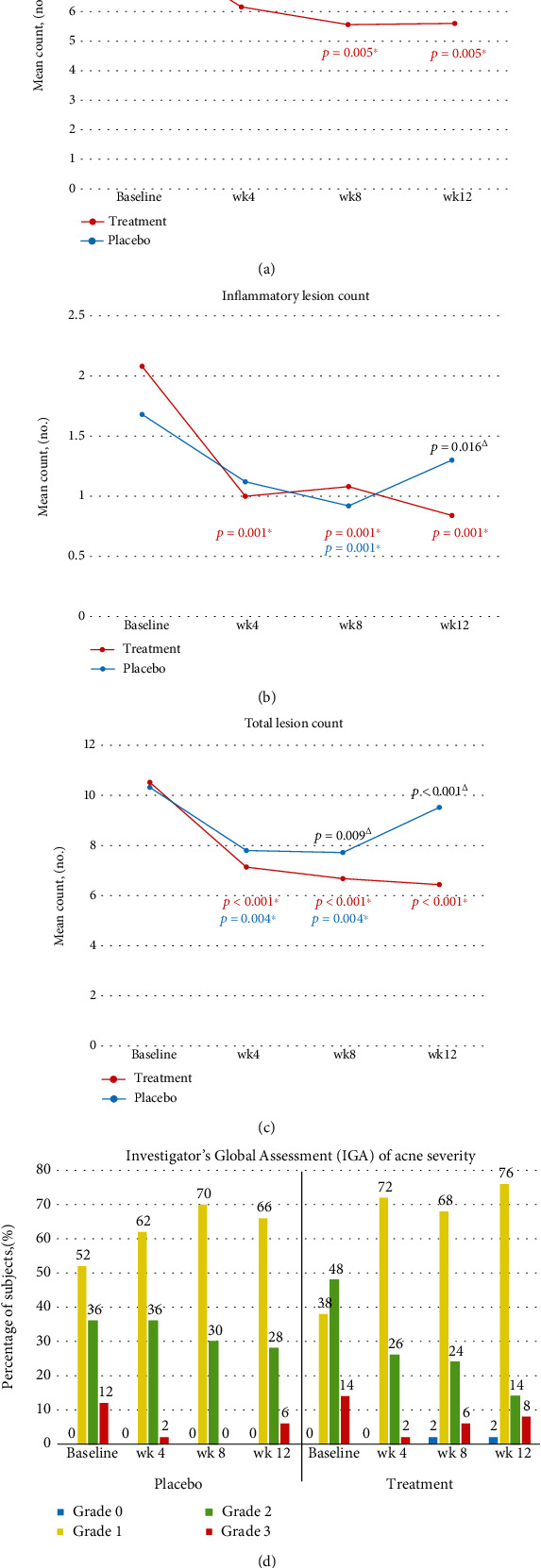
Comparison of (a) the mean noninflammatory lesion count, (b) inflammatory lesion count, (c) total lesion count, and (d) Investigator's Global Assessment of acne severity between the treatment side and placebo side at baseline and at weeks 4, 8, and 12. *^Δ^*Significant intergroup difference (*p* < 0.05). ^∗^Significant intragroup difference (*p* < 0.05).

After 12 weeks of maintenance therapy, the treatment group had a significant reduction in lesion count compared to the placebo group for each lesion type (noninflammatory, inflammatory, and total lesions), as shown in Figure 2. The mean values of noninflammatory count and total lesion count were significantly reduced at week 8 and week 12 compared to the placebo group (Figures 2(a) and 2(c), respectively), while the mean inflammatory lesion count was significantly reduced at week 12 (Figure 2(b)).

The treatment group had a significant reduction in mean noninflammatory, inflammatory, and total lesions from baseline to week 12 (Figures 2(a)–2(c), respectively). The placebo group also had a significant reduction in inflammatory lesion count from baseline to week 8 (Figure 2(b)) and in total lesion count from baseline to week 4 and from baseline to week 8 (Figure 2(c)). However, at the end of the study, lesion counts for all acne types rose substantially to nearly the same number of lesions counted at baseline in the placebo group (Figures 2(a)–2(c)). There was no significant reduction in the noninflammatory lesion count in the placebo group at any time point during the study period (Figure 2(a)).

Most patients in the treatment group shifted from grade 2 or 3 to grade 0 or 1, which is defined as clear or almost clear as assessed by IGA scoring (Figure 2(d)). The treatment group had a significantly higher mean total lesion count maintenance rate from baseline to week 12 (56%) than the placebo group (36%) (*p* = 0.021).

### 3.3. Skin Bioengineering Evaluation

Skin bioengineering evaluation data at each follow-up visit are shown in Table 2. Water content of the stratum corneum as assessed by Corneometer tended to increase from baseline to all time points in the treatment group. TEWL scores in both groups tended to decrease from baseline. However, none of the immediately aforementioned increases or decreases achieved statistical significance. In contrast, there was a significant reduction in skin sebum content in the treatment group compared to the placebo group at week 12.

### 3.4. Skin Tolerability Evaluation

The scores for the skin tolerability, including erythema, dryness, scaling, stinging/burning, and pruritus, are shown in Table 3. Patients evaluated erythema, dryness, scaling, sting/burning, and pruritus, whereas physicians evaluated erythema, dryness, and scaling. There were no significant differences in the mean scores for dryness, stinging/burning, or pruritus at any time point between treatment and placebo groups. Moreover, the treatment group had significantly less scaling than the placebo group at week 8. A more significant reduction in the mean erythema and scaling scores as assessed by the physicians were observed in the treatment group compared to the placebo group at week 12.

There was a significantly higher mean overall patient tolerance score in the treatment group than the placebo group at week 12 (*p* = 0.011). Most patients in the treatment group shifted their rating of overall patient tolerance from good at baseline to excellent at week 12. No serious or severe adverse events were reported.

### 3.5. Skin Radiance Evaluation

Skin radiance as assessed by a patient-rated VAS score increased from baseline in both groups. However, the treatment group had a significantly higher mean score than the placebo group at week 12 (*p* = 0.014).

### 3.6. Rating of Patient and Physician Satisfaction

The treatment group had a higher mean patient satisfaction score than the placebo group at all time points. Most patients in the treatment group shifted their rating of satisfaction from moderate at baseline to very satisfied at week 12. In addition, the treatment group had a significantly higher mean VAS score for patient satisfaction than the placebo group at week 12 (*p* = 0.009).

The treatment group had a significantly higher mean physician satisfaction score than the placebo group at week 12 (*p* < 0.001). Most patients in the treatment group shifted the rating by physician assessment from good at the baseline towards good and excellent at week 12. Moreover, the treatment group had a significantly higher mean VAS score for physician satisfaction than the placebo group at week 12 (*p* < 0.001).

### 3.7. Photographic Evaluation

**Figure 3:**
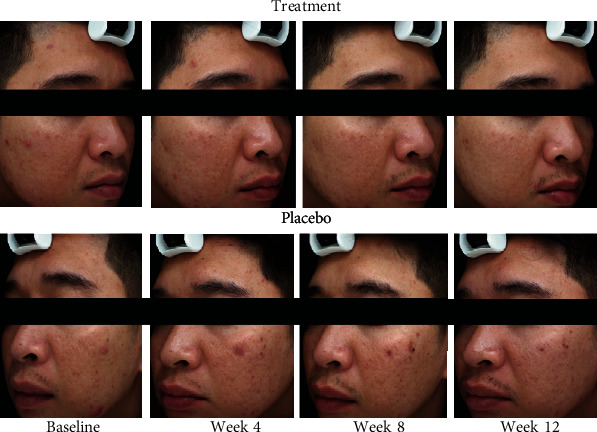
Representative photographs of an acne vulgaris patient compared between the treatment side and the placebo side of the face at baseline and at weeks 4, 8, and 12. The treatment side had better clinical response than the placebo side.

**Figure 4:**
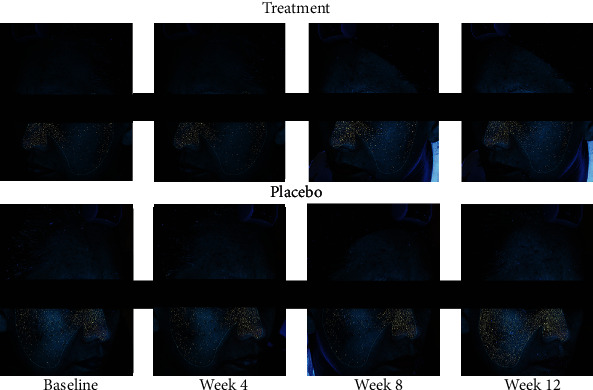
Digital ultraviolet fluorescence photography comparing *Cutibacterium acnes* (*C. acnes*) between the treatment side and the placebo side of the face at baseline and at weeks 4, 8, and 12. The treatment side had a much lower amount of *C. acnes* than the placebo side.

The clinical response of one patient at baseline, week 4, week 8, and week 12 is shown in Figure 3. The treatment side showed better clinical response than the placebo side. Digital UV fluorescence photography showed a marked reduction in porphyrins excreting from *C. acnes* in the treatment group compared to the placebo group at week 12 (Figure 4).

## 4. Discussion

Maintenance therapy after successful induction treatment might be beneficial for preventing lesions from relapsing by suppressing the development of microcomedones and by targeting *C. acnes* colonization [3]. Dermocosmetics are being increasingly used in the long-term treatment of acne vulgaris [11]. Angelova-Fischer et al. [13] showed that moisturizer containing licochalcone A, L-carnitine, and 1,2-decanediol efficiently improved mild to moderate severe acne compared to a vehicle cream. Chularojanamontri et al. [14] reported that a moisturizer containing these ingredients could encourage patient adherence and improve unfavorable adverse effects, such as irritation. Dall'Oglio et al. [15] suggested that the daily licochalcone A moisturizer with salicylic acid/L-carnitine as fluid or with hydroxy complex 10% as cream was used as an adjunctive treatment for mild acne. Recently, Wanitphakdeedecha et al. [16] reported that a moisturizer containing the active ingredients of licochalcone A, L-carnitine, 1,2-decanediol, and salicylic acid, which are the same ingredients as those used in our study moisturizer, provided good synergistic effect when combined with photodynamic therapy to treat acne.

The present study demonstrated that moisturizer containing licochalcone A, L-carnitine, 1,2-decanediol, and salicylic acid could maintain the clinical response produced by 8 weeks of induction phase treatment for acne. The treatment group had a significant reduction in noninflammatory, inflammatory, and total lesions at the end of the study and a significantly higher maintenance rate for total lesions compared to the placebo group.

Table 4 shows data from previously published randomized controlled studies of the maintenance phase of acne therapy compared to the present study. Our result demonstrated that the treatment group had a significantly higher maintenance rate for mean total lesions (56%) compared to the placebo group (36%). Among 5 previous studies of maintenance phase of acne therapy [8, 23, 25, 26, 31], Thielitz et al. [8] recruited patients with mild to moderate acne whereas the others enrolled patients with moderate to severe acne. The maintenance rate in the treatment group in our study in patients with mild to moderate acne using dermocosmetic as monotherapy was slightly lower than the rate using adapalene in the study of Thielitz et al. [8]

Various active ingredients may lead to favorable acne control outcomes. Licochalcone A is an extract of *Glycyrrhiza inflata*, which has anti-inflammatory and antimicrobial properties. An *in vivo* study demonstrated its anti-irritative effects, including a significant decrease in erythema in shave-related and UV-induced erythema procedures [17]. 1,2-Decanediol was shown to reduce the growth of *C. acnes*, and it inhibited *C. acnes* biofilm formation [18]. Increased sebum production is one of the main pathogenic factors that contribute to the development of acne lesions [32]. L-Carnitine was recently demonstrated to decrease sebum secretion via stimulation of *β*-oxidation and reduced intracellular lipid content in human sebocytes [19]. Salicylic acid is a beta-hydroxy acid that has keratolytic, comedolytic, and anti-inflammatory properties, and it was shown to have favorable effect on both noninflammatory and inflammatory acne lesions [20, 21].

A corneometer was used to evaluate skin surface hydration [33]. In our study, the treatment group had slight improvement in skin hydration compared to baseline, but the placebo group did not. However, there was no statistically significant difference. TEWL is the amount of water that passively evaporates through the skin to the external environment due to water vapor pressure gradient on both sides of the skin barrier. The measurement of TEWL or skin surface vapor loss is a good indicator of the integrity of skin barrier function or the skin's ability to retain moisture. TEWL in both groups in this study tended to decrease from baseline. This might be explained by the properties of the vehicle that was used in both the treatment and placebo moisturizers. However, sebumeter measurement showed sebum production in patients in the treatment group to be significantly lower than the sebum production in the placebo group at week 12. This result supports that the treatment solution can reduce sebum production due to the property of L-carnitine. Satisfaction assessment by both patients and physicians showed significantly greater satisfaction with the moisturizer containing the active ingredients than the placebo moisturizer at the end of the study.

### 4.1. Study Limitations

This study included only patients with mild to moderate acne severity. Further studies should evaluate the efficacy of the studied active ingredients as maintenance therapy in patients with more severe acne, in a larger population, and for a longer period of time.

## 5. Conclusions

This study demonstrated the efficacy and safety of moisturizer containing licochalcone A, decanediol, L-carnitine, and salicylic acid as maintenance therapy in Thai patients with mild to moderate severity acne, which might represent the Asian population. This moisturizer could reduce the development of new acne lesions, as well as prevent acne relapse.

## Figures and Tables

**Table 1:** Baseline characteristics compared between the treatment side and placebo side (split-face study) during the maintenance phase (*N* = 50).

Variables	Mean ± SD or *N* (%)		*p* value
	Treatment side	Placebo side	
Clinical characteristics			
Acne lesion counts			
Noninflammatory	8.4 ± 5.8	8.7 ± 5.5	0.684
Inflammatory	2.1 ± 1.9	1.7 ± 1.3	0.060
Total	10.5 ± 6.5	10.3 ± 5.9	0.766
IGA scale for acne vulgaris			
Grade 1 (almost clear)	19 (38%)	26 (52%)	0.147
Grade 2 (mild severity)	24 (48%)	18 (36%)	
Grade 3 (moderate severity)	7 (14%)	6 (12%)	
Skin bioengineering measurements			
Corneometer (corneometer unit)	70.4 ± 13.0	69.6 ± 12.9	0.164
Tewameter (g/h/m^2^)	13.2 ± 6.8	13.9 ± 8.5	0.463
Sebumeter (*μ*g/cm^2^)	41.2 ± 24.4	40.4 ± 25.9	0.603

A *p* value < 0.05 indicates statistical significance. SD: standard deviation; IGA: Investigator's Global Assessment.

**Table 2:** Skin bioengineering evaluation compared between the treatment side and placebo side (split-face study) during the maintenance phase (*N* = 50).

Variables	Mean ± SD		*p* value
	Treatment side	Placebo side	
Skin hydration (corneometer unit)			
Baseline	70.4 ± 13.0	69.6 ± 12.9	0.164
Week 4	71.8 ± 10.9	69.4 ± 13.3	0.140
Week 8	72.9 ± 11.0	70.7 ± 11.8	0.174
Week 12	71.2 ± 12.1	69.3 ± 13.2	0.237
Transepidermal water loss (g/h/m^2^)			
Baseline	13.2 ± 6.8	13.9 ± 8.5	0.463
Week 4	11.7 ± 8.6	9.9 ± 4.4	0.166
Week 8	10.2 ± 5.7	10.9 ± 5.0	0.197
Week 12	10.1 ± 5.1	10.6 ± 5.4	0.415
Sebumeter (*μ*g/cm^2^)			
Baseline	41.2 ± 24.4	40.4 ± 25.9	0.603
Week 4	39.9 ± 26.1	43.3 ± 22.7	0.147
Week 8	37.7 ± 25.0	40.8 ± 28.4	0.111
Week 12	35.3 ± 26.4	39.7 ± 27.9	0.029^∗^

^∗^A *p* value < 0.05 indicates statistical significance. SD: standard deviation.

**Table 3:** Tolerability assessment compared between the treatment side and placebo side (split-face study) during the maintenance phase (*N* = 50).

Variables	Mean ± SD		*p* value
	Treatment side	Placebo side	
*Patient evaluation*			
Erythema (score = 0-3)			
Baseline	1.6 ± 0.8	1.6 ± 0.8	0.554
Week 4	1.1 ± 0.8	1.2 ± 0.8	0.659
Week 8	0.9 ± 0.8	1.0 ± 0.8	0.278
Week 12	0.9 ± 0.9	1.0 ± 0.8	0.351
Dryness (score = 0-3)			
Baseline	1.4 ± 1.1	1.5 ± 0.9	0.212
Week 4	1.0 ± 0.9	1.0 ± 0.9	0.497
Week 8	0.8 ± 0.8	0.9 ± 0.9	0.133
Week 12	0.8 ± 0.9	0.9 ± 0.9	0.444
Scaling (score = 0-3)			
Baseline	1.1 ± 0.9	1.08 ± 0.9	1.000
Week 4	0.4 ± 0.6	0.4 ± 0.7	1.000
Week 8	0.4 ± 0.6	0.7 ± 0.8	0.001^∗^
Week 12	0.5 ± 0.7	0.6 ± 0.8	0.622
Stinging/burning (score = 0-3)			
Baseline	0.7 ± 0.7	0.8 ± 0.7	0.766
Week 4	0.4 ± 0.8	0.3 ± 0.6	0.204
Week 8	0.5 ± 0.7	0.6 ± 0.9	0.135
Week 12	0.2 ± 0.5	0.3 ± 0.6	0.485
Pruritus (score = 0-3)			
Baseline	0.7 ± 0.8	0.7 ± 0.7	1.000
Week 4	0.3 ± 0.6	0.7 ± 0.5	0.371
Week 8	0.4 ± 0.7	0.4 ± 0.6	1.000
Week 12	0.3 ± 0.6	0.3 ± 0.5	1.000
*Physician evaluation*			
Erythema (score = 0-3)			
Baseline	1.8 ± 0.6	1.9 ± 0.7	0.302
Week 4	1.3 ± 0.6	1.4 ± 0.6	0.110
Week 8	1.2 ± 0.6	1.3 ± 0.6	0.182
Week 12	1.0 ± 0.6	1.2 ± 0.4	0.044^∗^
Dryness (score = 0-3)			
Baseline	1.0 ± 0.6	1.0 ± 0.6	0.569
Week 4	0.7 ± 0.5	0.7 ± 0.5	1.000
Week 8	0.6 ± 0.5	0.6 ± 0.6	0.322
Week 12	0.6 ± 0.6	0.6 ± 0.7	0.420
Scaling (score = 0-3)			
Baseline	0.6 ± 0.7	0.6 ± 0.7	0.322
Week 4	0.2 ± 0.5	0.2 ± 0.4	1.000
Week 8	0.3 ± 0.5	0.3 ± 0.8	0.420
Week 12	0.3 ± 0.5	0.5 ± 0.7	0.005^∗^

^∗^A *p* value < 0.05 indicates statistical significance. Scoring: 0 = none, 1 = mild, 2 = moderate, and 3 = severe. *N*: number; SD: standard deviation.

**Table 4:** Literature review of randomized controlled studies that investigated maintenance therapy for acne compared with that of the present study.

	Thiboutot et al. [34], 2005; Thiboutot et al. [31], 2006	Leyden et al. [23], 2006	Thielitz et al. [8], 2007	Alirezai et al. [26], 2007	Gold et al. [24], 2010; Poulin et al. [25], 2011	The present study, 2020
Induction phase						
Number of patients	467	189	54	242	459	110
Acne severity	Severe	Moderately severe to severe	Mild to moderate	Moderate to moderately severe	Severe	Mild to moderate
Treatment	Adapalene gel 0.1% + doxycycline 100 mg versus doxycycline 100 mg + vehicle	Tazarotene gel 0.1% + minocycline 100 mg	Adapalene 0.1% + BPO 2.5%gel	Lymecycline 300 mg + adapalene gel 0.1% versus lymecycline 300 mg + vehicle	Doxycycline 100 mg + adapalene-BPO 2.5% gel versus doxycycline 100 mg + vehicle	Fixed combination of adapalene 0.1% and BPO 2.5%gel ± doxycycline100-200 mg(moderate cases)
Duration	12 weeks	12 weeks	8 weeks	12 weeks	12 weeks	8 weeks
Maintenance phase						
Definition of responders	At least 50% improvement from baseline	At least 75% improvement from baseline	Subjects who successfully completed the combination phase entered the 12-week maintenance phase	At least moderate improvement from baseline	At least 50% global improvement after a treatment phase	At least 50% reduction in lesion counts or at least 2-grade improvements according to IGA score from baseline
Number of patients	253	110	49	136	243	50 (split-face study)
Treatment	Adapalene gel 0.1% versus vehicle	Tazarotene gel 0.1% + placebo capsules versus vehicle gel + minocycline capsules versus tazarotene gel 0.1% + minocycline capsules	Adapalene daily versus adapalene in alternation with its vehicle versus vehicle	Adapalene gel 0.1% versus vehicle	Adapalene-BPO 2.5% gel versus vehicle	Moisturizer with and without the active ingredients containing of licochalcone A, 1,2-decanediol, L-carnitine, and salicylic acid
Duration	16 weeks	12 weeks	12 weeks	12 weeks	24 weeks	12 weeks
Maintenance rate^∗^	Adapalene maintenance therapy resulted in significantly larger maintenance rate of total lesions compared with vehicle (75% *vs.* 54%; *p* < 0.01)	At week 12, more than 80% of patients in each group had maintained a 50% improvement in total lesions from baseline with no significance	Adapalene daily maintenance therapy resulted in higher maintenance rate of total lesions (68% *vs*. 59% *vs*. 41%), however no statistical significance	Adapalene maintenance therapy resulted in significantly larger maintenance rate of total lesions compared with vehicle (84.7% *vs.* 63.5%; *p* = 0.0049)	At week 24, adapalene-BPO gel resulted in significantly higher maintenance rate for total lesions (78% *vs.* 45.8%; *p* < 0.001)	The treatment group had a significantly higher maintenance rate for mean total lesion count between baseline and week 12 (56%) compared to the vehicle group (36%) (*p* = 0.021)

^∗^Maintenance rate was defined as the percentage of patients who maintained at least 50% improvement in the total lesion count when entering the maintenance phase from baseline to the end of the study. BPO: benzoyl peroxide; IGA: Investigator's Global Assessment.

## Data Availability

The data used to support the findings of this study are available from the corresponding author upon request.

## Supplementary Materials

Supplementary Materials CONSORT 2010 checklist for randomized controlled trials.
