# Supplementary material for: A Double-Blinded, Randomized, Vehicle-Controlled Study of the Efficacy of Moisturizer Containing Licochalcone A, Decanediol, L-Carnitine, and Salicylic Acid for Prevention of Acne Relapse in Asian Population
Source: Biomed Res Int. 2020 Oct 16;2020:2857812. doi: 10.1155/2020/2857812 (PMC7603542; doi:10.1155/2020/2857812)
Supplement: Supplementary Materials — CONSORT 2010 checklist for randomized controlled trials. [file 2857812.f1.doc]

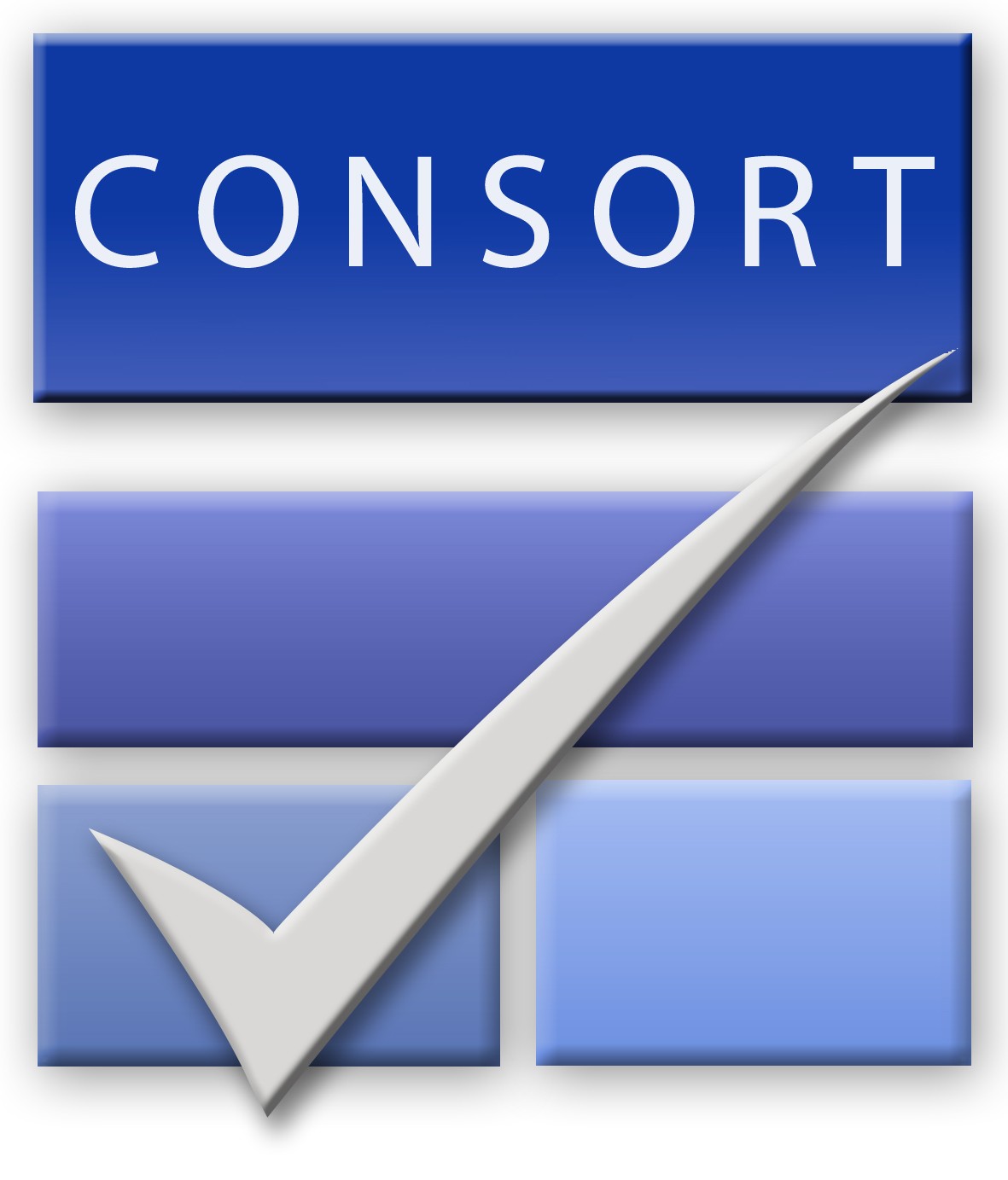
CONSORT 2010 checklist of information to include when reporting a randomised trial*

| Section/Topic | Item No | Checklist item | Reported on page No |
| --- | --- | --- | --- |
| Title and abstract | | | |
|  | 1a | Identification as a randomised trial in the title | Page No. 2 (line 28) |
|  | 1b | Structured summary of trial design, methods, results, and conclusions (for specific guidance see CONSORT for abstracts) | Page No. 2 (line 23-38) |
| Introduction | | | |
| Background and objectives | 2a | Scientific background and explanation of rationale | Page No. 3-4 (line 40-65) |
|  | 2b | Specific objectives or hypotheses | Page No. 4 (line 67-70) |
| Methods | | | |
| Trial design | 3a | Description of trial design (such as parallel, factorial) including allocation ratio | Page No. 5-6 (line 111-131) |
|  | 3b | Important changes to methods after trial commencement (such as eligibility criteria), with reasons 🡪 No change | Page No. 6 (line 132-134) |
| Participants | 4a | Eligibility criteria for participants | Page No. 4-5 (line 80-92) |
|  | 4b | Settings and locations where the data were collected | Page No. 4 (line 74-75) |
| Interventions | 5 | The interventions for each group with sufficient details to allow replication, including how and when they were actually administered | Page No. 5-6 (line 111-136) |
| Outcomes | 6a | Completely defined pre-specified primary and secondary outcome measures, including how and when they were assessed | Page No. 7-9 (line 139-180) |
|  | 6b | Any changes to trial outcomes after the trial commenced, with reasons 🡪 No change | Page No. 6 (line 134-136) |
| Sample size | 7a | How sample size was determined | Page No. 5 (line 94-109) |
|  | 7b | When applicable, explanation of any interim analyses and stopping guidelines | Page No. 6 (line 136) |
| Randomisation: |  |  |  |
| Sequence generation | 8a | Method used to generate the random allocation sequence | Page No. 6 (line 125) |
|  | 8b | Type of randomisation; details of any restriction (such as blocking and block size) | Page No. 6 (line 126) |
| Allocation concealment mechanism | 9 | Mechanism used to implement the random allocation sequence (such as sequentially numbered containers), describing any steps taken to conceal the sequence until interventions were assigned | Page No. 6 (line 126-131) |
| Implementation | 10 | Who generated the random allocation sequence, who enrolled participants, and who assigned participants to interventions | Page No. 6 (line 132) |
| Blinding | 11a | If done, who was blinded after assignment to interventions (for example, participants, care providers, those assessing outcomes) and how | Page No. 6 (line 125-132) |
|  | 11b | If relevant, description of the similarity of interventions | Page No. 6 (line 125-132) |
| Statistical methods | 12a | Statistical methods used to compare groups for primary and secondary outcomes | Page No. 9 (line 184-187) |
|  | 12b | Methods for additional analyses, such as subgroup analyses and adjusted analyses | Not available |
| Results | | | |
| Participant flow (a diagram is strongly recommended) | 13a | For each group, the numbers of participants who were randomly assigned, received intended treatment, and were analysed for the primary outcome | Page No. 7 (line 137) and  Page No. 9 (line 192-195) |
|  | 13b | For each group, losses and exclusions after randomisation, together with reasons | Page No. 9 (line 195-196) |
| Recruitment | 14a | Dates defining the periods of recruitment and follow-up | Page No. 4 (line 75) |
|  | 14b | Why the trial ended or was stopped | Page No. 9 (line 195-196) |
| Baseline data | 15 | A table showing baseline demographic and clinical characteristics for each group | Page No. 9 (line 197) |
| Numbers analysed | 16 | For each group, number of participants (denominator) included in each analysis and whether the analysis was by original assigned groups | Page No. 9 (line 197) |
| Outcomes and estimation | 17a | For each primary and secondary outcome, results for each group, and the estimated effect size and its precision (such as 95% confidence interval) | Page No. 10-16 (line 206-276) |
|  | 17b | For binary outcomes, presentation of both absolute and relative effect sizes is recommended | Not available |
| Ancillary analyses | 18 | Results of any other analyses performed, including subgroup analyses and adjusted analyses, distinguishing pre-specified from exploratory | Not available |
| Harms | 19 | All important harms or unintended effects in each group (for specific guidance see CONSORT for harms) | Page No. 13-14 (line 242-254) |
| Discussion | | | |
| Limitations | 20 | Trial limitations, addressing sources of potential bias, imprecision, and, if relevant, multiplicity of analyses | Page No. 21 (line 358-361) |
| Generalisability | 21 | Generalisability (external validity, applicability) of the trial findings | Page No. 21 (line 366) |
| Interpretation | 22 | Interpretation consistent with results, balancing benefits and harms, and considering other relevant evidence | Page No. 18-20 (line 316-357) |
| Other information | | |  |
| Registration | 23 | Registration number and name of trial registry | Page No. 4 (line 76) |
| Protocol | 24 | Where the full trial protocol can be accessed, if available | Page No. 7 (line 137) |
| Funding | 25 | Sources of funding and other support (such as supply of drugs), role of funders | Page No. 21 (line 381) |

*We strongly recommend reading this statement in conjunction with the CONSORT 2010 Explanation and Elaboration for important clarifications on all the items. If relevant, we also recommend reading CONSORT extensions for cluster randomised trials, non-inferiority and equivalence trials, non-pharmacological treatments, herbal interventions, and pragmatic trials. Additional extensions are forthcoming: for those and for up to date references relevant to this checklist, see [www.consort-statement.org](http://www.consort-statement.org/).
